# Effect of maternal smoking during pregnancy on gestational weight gain and birthweight: A stratified analysis by pregestational weight status

**DOI:** 10.18332/tid/143952

**Published:** 2022-01-28

**Authors:** Miho Suzuki, Rei Wakayama, Zentaro Yamagata, Kohta Suzuki

**Affiliations:** 1 Nagoya Bunri Nutrition College, Nagoya, Japan; 2 Graduate School of Human Life Science, Osaka City University, Osaka, Japan; 3 Department of Health and Psychosocial Medicine, Aichi Medical University School of Medicine, Nagakute, Japan; 4 Department of Health Sciences, Graduate School of Medical Science, University of Yamanashi, Chuo, Japan

**Keywords:** gestational weight gain, low birthweight, maternal smoking, pregnancy, nutrition

## Abstract

**INTRODUCTION:**

In Japan, low birthweight (LBW) of infants is a major public health concern. Gestational weight gain (GWG) may be associated with LBW of infants. On the other hand, maternal smoking during pregnancy is a well-known factor associated with infant birthweight. Thus, this study aimed to examine the effect of maternal smoking during pregnancy on GWG and birthweight stratified by pregestational weight status.

**METHODS:**

A retrospective cohort study was designed, which included three hospitals of Yamanashi Prefecture, Japan. The study included babies born between 2013 and 2014 from mothers having singleton pregnancies. The outcomes analyzed were GWG (difference between maternal weight measured at prenatal check-up just before delivery and pregestational weight based on information from clinical records) and birthweight of infants stratified by pregestational maternal body mass index (BMI).

**RESULTS:**

This study included 1150 singleton babies and their mothers. After excluding individuals with incomplete data, the final number of analyzed participants was 1078. The mean maternal age at delivery was 31.3 ± 5.1 years. The mean pregestational BMI was 21.3 ± 3.4 kg/m^2^. The mean GWG was 10.0 ± 4.1 kg. After adjusting for confounding factors, maternal smoking during pregnancy was positively associated with GWG in all categories (underweight: p<0.0001; normal weight: p<0.0001; overweight: p=0.01). Maternal smoking during pregnancy was also significantly associated with lower birthweight in underweight and normal-weight mothers (underweight: p=0.04, normal-weight: p=0.03).

**CONCLUSIONS:**

Maternal smoking status is significantly associated with higher GWG and lower birthweight. Based on the Developmental Origins of Health and Disease (DOHaD) hypothesis, the growth of infants born from smoking mothers needs close observation.

## INTRODUCTION

In Japan, the prevalence of low birthweight (LBW) infants is relatively higher than in other countries. Moreover, the prevalence has increased from the 1970s. For example, 5.5% of female infants born in 1975 weighed <2500 g, which has increased to 10.6% in 2016^1^. In recent years, the Developmental Origins of Health and Diseases (DOHaD) hypothesis has been proposed in addition to the established ‘fetal programming’ hypothesis and ‘Barker's hypothesis’, to support the mechanisms of childhood growth and development^2^. These hypotheses describe how a specific growth trajectory, such as slow fetal growth and subsequent rapid infant growth, is associated with the development of chronic diseases in adulthood^2-6^. Thus, as significantly described by these hypotheses, appropriate fetal growth is an important factor for the future health of an individual.

Several risk factors for LBW were identified, including maternal smoking during pregnancy^7-9^ and low socioeconomic status^10-12^. In addition, insufficient gestational weight gain (GWG) is considered a risk factor for LBW as well as maternal smoking during pregnancy^13,14^. In 2006, the Japanese Ministry of Health, Labor and Welfare established the guidelines for GWG stratified by pregestational maternal body mass index (BMI, kg/m^2^), termed ‘Dietary guidelines for maternity’^15^. As reported, the percentages of women with GWG below the recommended range were 32% for underweight (BMI <18.5), 14% for normal weight (18.5≤ BMI <24.9), and 46% for overweight (BMI ≥25.0) women^16^. Given that lower GWG and maternal smoking during pregnancy are significantly associated with small-for-gestationalage (SGA) infants, it is recommended that pregnant women in Japan improve their GWG^17^. In this regard, the incidence of SGA infants in underweight women is not significantly increased if these women have appropriate GWG^18^.

However, given the paucity of information on factors associated with GWG, it is necessary to clarify the current status of GWG and identify factors associated with it. We also need to consider the effect of smoking during pregnancy on GWG and birthweight. Thus, the primary aim of this study was to examine the effect of maternal smoking during pregnancy on GWG stratified by the pregestational BMI using prenatal check-up data. The secondary aim was to examine the effect of maternal smoking during pregnancy and related factors, including GWG, with the birthweight of infants.

## METHODS

### Study design

This multiple hospital-based retrospective cohort study based on the medical records was conducted in two community hospitals and one university hospital in Yamanashi Prefecture, Japan. Participants were singleton babies born between the following periods: Yamanashi Red Cross Hospital, 1 June 2013 to 31 March 2014; Fujiyoshida Municipal Hospital, 1 August 2013 to 31 March 2014; and University of Yamanashi Hospital, 1 January 2013 to 30 June 2014; and their mothers. Thus, every patient who met the above inclusion criteria and had prenatal check-ups participated in the study. There was no other inclusion and exclusion criterion for study participant selection.

Data collecting staff in each hospital referred the clinical records and extracted essential information. Because part of the data were not based on electronical database, the staff transferred the information from the paper records.

Regarding the sample size calculation, we referred to our previous study^8,9^. Based on a smoking rate of 5% and the response within each subject group was normally distributed with a weight standard deviation of 400 g, and if the true difference in the smoking group and control means is 120 g, then 92 smoking subjects and 1840 non-smoking subjects are needed to reject the null hypothesis that the population means of the experimental and control groups are equal with probability (power) 0.8. The Type I error probability associated with this test of this null hypothesis is 0.05. However, because this study was conducted using limited resources, including data-collecting staff, it was difficult to collect the number of participants and the study periods differed for each hospital.

### Data collection

All data were obtained from the clinical records of prenatal check-ups. The primary outcome was GWG, defined as the difference between maternal weight measured at the prenatal check-up just before delivery, and pregestational weight based on information from clinical records. Data regarding the following maternal variables were collected from clinical records: pregnancy history (primipara or multipara), maternal age (<25, 25–34 or ≥35 years), pregestational maternal BMI (underweight <18.5; normal weight 18.5–24.9, overweight ≥25 kg/m^2^), and maternal smoking during pregnancy (smoker or never/non-smoker). Information on birthweight and gestational age at birth was also collected from medical records. Smoker was defined based on the description of these records.

### Statistical analysis

Characteristics of mothers and their babies were stratified by maternal pregestational weight status based on BMI. Multiple linear regression models for GWG (primary outcome) and birthweight (secondary outcome) stratified by pregestational weight status were used to examine the effects of maternal and infant variables on these outcomes. All statistical analyses were performed using STATA version 15 (StataCorp LLC, College Station, TX). Two-sided p<0.05 was considered statistically significant.

## RESULTS

### Characteristics of participants

The total number of singleton babies and their mothers was 1150. From this sample, we only used data that included the following complete information: sex of the child, maternal age at delivery, gestational age at delivery, maternal height and weight before pregnancy, and maternal weight measured at the prenatal check-up just before delivery. After excluding those without complete data, 1078 participants were analyzed; they were 573 (53.2%), 214 (19.9%) and 291 (27.0%) from the Yamanashi Red Cross Hospital, Fujiyoshida Municipal Hospital, and University of Yamanashi Hospital, respectively. There were 519 (48.1%) primiparous mothers. The mean maternal age at delivery was 31.3 ± 5.1 years. The mean pregestational BMI was 21.3 ± 3.4 kg/m^2^. The mean GWG was 10.0 ± 4.1 kg. Information on smoking status was obtained from 1033 mothers, and 109 (10.6%) mothers were smokers during pregnancy. There were 553 (51.3%) male children. The mean birthweight and mean gestational duration were 2984 ± 381.5 g and 273.8 ± 9.3 days, respectively (Table 1).

**Table 1:** Baseline characteristics of participants, Japan, 2013–2014 (N=1150)

*Characteristics*	*Total*		*Underweight (BMI <18.5)*		*Normal weight (18.5≤ BMI <25)*		*Overweight (BMI ≥25)*	
	*n or n (%)*	*Mean±SD*	*n or n (%)*	*Mean±SD*	*n or n (%)*	*Mean±SD*	*n or n (%)*	*Mean±SD*
**Maternal age at delivery** (years)	1078	31.3±5.1	177	30.3±5.1	762	31.2±5.0	139	32.7±5.5
**Pregestational height** ( cm )	1078	158.0±5.3	177	158.7±5.4	762	157.9±5.2	139	157.5±5.5
**Pregestational weight** (kg)	1078	53.3±9.0	177	44.2±3.6	762	52.4±5.2	139	69.7±8.9
**Pregestational maternal BMI** (kg/m^2^)	1078	21.3±3.4	177	17.5±0.8	762	21.0±1.6	139	28.1±3.2
**Gestational period** ( days )	1078	273.8±9.3	177	273.3±9.7	762	274.2±9.0	139	273±10
**Weight gain during pregnancy** (kg)	1078	10.0±4.1	177	10.5±3.7	762	10.4±3.8	139	7.3±5.0
**Birthweight** (g)	1078	2984±381.5	177	2893.7±391.8	762	2992.9±371.4	139	3050.2±405.4
**Infant sex**	1078		177		762		139	
Male	553 (51.3)		92 (52.0)		389 (51.1)		72 (51.8)	
Female	525 (48.7)		85 (48.0)		373 (49.0)		67 (48.2)	
**Parity**	1078		177		762		139	
Primiparity	519 (48.1)		98 (55.4)		357 (46.9)		64 (46.0)	
Multiparity	559 (51.9)		79 (44.6)		405 (53.2)		75 (54.0)	
**Smoking during pregnancy**	1.033		174		726		133	
Non-smokers	924 (89.4)		156 (89.7)		654 (90.1)		114 (85.7)	
Current smokers	109 (10.6)		18 (10.3)		72 (9.9)		19 (14.3)	

BMI: body mass index. SD: standard deviation.

Characteristics of the participants are described in Table 1. The numbers of underweight, normal-weight, and overweight participants were 177 (16.4%), 762 (70.7%), and 139 (12.9%), respectively. The mean maternal ages of underweight, normal-weight, and overweight participants were 30.3 ± 5.1, 31.2 ± 5.0, and 32.7 ± 5.5 years, respectively. The mean GWG of underweight, normal-weight, and overweight participants were 10.5 ± 3.7, 10.4 ± 3.8, and 7.3 ± 5.0 kg, respectively. The mean birthweights of infants born from underweight, normal-weight, and overweight mothers were 2893.7 ± 391.8, 2992.9 ± 371.4, and 3050.2 ± 405.4 g, respectively.

### Multiple linear regression models

After adjusting for confounding factors, including gestational duration, pregnancy history, and gender of infants, pregestational BMI was negatively associated with GWG in normal-weight and overweight mothers (normal weight: b= -0.20, p=0.02; overweight: b= -0.90, p<0.0001). Maternal smoking during pregnancy was positively associated with GWG in all categories (underweight: b=3.81, p<0.0001; normal-weight: b=2.45, p<0.0001; overweight: b=2.78, p=0.01) (Table 2).

**Table 2:** Multiple linear regression analysis of weight change during pregnancy with respect to pregestational maternal body mass index, Japan, 2013–2014 (N=1150)

*Variables*	*Underweight (BMI <18.5)*			*p*	*Normal weight (18.5≤ BMI <25)*			*p*	*Overweight (BMI ≥25)*			*p*
	*b*	*S.E.*	*t*		*b*	*S.E.*	*t*		*b*	*S.E.*	*t*	
**Intercept**	-14.92	8.78	-1.70	0.09	-12.12	4.45	-2.72	0.007	-2.47	11.49	-0.21	0.8
**Age of mother at pregnancy registration** ( years )				0.2*				0.048*				0.2*
Age <25	0.39	0.83	0.46	0.6	0.93	0.50	1.87	0.06	-3.25	1.87	-1.74	0.08
25 ≤ Age <35 (Ref.)												
Age ≥35	-1.14	0.67	-1.69	0.09	-0.42	0.32	-1.31	0.2	-0.92	0.80	-1.15	0.3
**Pregestational BMI**	-0.29	0.35	-0.84	0.4	-0.20	0.08	-2.45	0.02	-0.90	0.15	-6.20	<0.0001
**Gestational period**	0.11	0.03	4.06	<0.0001	0.10	0.02	6.48	<0.0001	0.13	0.04	3.20	0.002
**Infant sex**												
Male (Ref.)												
Female	0.25	0.52	0.48	0.6	-0.12	0.27	-0.44	0.7	0.05	0.78	0.07	0.9
**Parity**												
Primiparity (Ref.)												
Multiparity	-0.33	0.54	-0.62	0.5	-0.25	0.28	-0.89	0.4	0.65	0.79	0.83	0.4
**Smoking during pregnancy**												
Non-smokers (Ref.)												
Current smokers	3.81	0.87	4.39	<0.0001	2.45	0.45	5.49	<0.0001	2.78	1.09	2.54	0.01

* Three-category variables were tested by F-test. BMI: body mass index.

After adjusting for confounding factors, GWG was positively associated with birthweight among underweight and normal-weight mothers (underweight: b=22.18, p=0.001; normal-weight: b=19.41, p<0.0001). Maternal smoking during pregnancy was also significantly associated with lower birthweight in underweight and normal-weight mothers (underweight: b= -163.13, p=0.04; normal-weight: b= -85.07, p=0.03) (Table 3).

**Table 3:** Multiple linear regression analysis of birthweight with respect to pregestational maternal body mass index, Japan, 2013–2014 (N=1150)

*Variables*	*Underweight (BMI <18.5)*			*p*	*Normal weight (18.5≤ BMI <25)*			*p*	*Overweight (BMI ≥25)*			*p*
	*b*	*S.E.*	*t*		*b*	*S.E.*	*t*		*b*	*S.E.*	*t*	
**Intercept**	-2895.30	776.23	-3.73	<0.0001	-3144.15	377.98	-8.32	<0.0001	-3624.71	878.03	-4.13	<0.0001
**Weight gain during pregnancy**	22.18	6.80	3.26	0.001	19.41	3.15	6.16	<0.0001	10.93	6.83	1.60	0.1
**Age of mother at pregnancy registration** ( years )				0.2*				0.7*				0.8*
Age <25	-104.92	72.76	-1.44	0.2	-0.50	42.15	-0.01	1.0	0.71	144.19	0.00	1.0
25 ≤ Age <35 (Ref.)												
Age ≥35	-86.54	59.39	-1.46	0.1	24.09	26.98	0.89	0.4	-41.60	61.31	-0.68	0.5
**BMI**	-10.52	-10.52	-0.34	0.7	18.78	6.91	2.72	0.007	8.27	12.72	0.65	0.5
**Gestational period**	21.20	2.51	8.44	<0.0001	20.28	1.31	15.50	<0.0001	23.44	3.15	7.45	<0.0001
**Infant sex**												
Male (Ref.)												
Female	-156.40	45.63	-3.43	0.001	-125.85	22.67	-5.55	<0.0001	-104.00	59.64	-1.74	0.08
**Parity**												
First birth (Ref.)												
Second or more	137.59	47.16	2.92	0.004	83.62	23.37	3.58	<0.0001	84.28	60.33	1.40	0.2
**Smoking during pregnancy**												
Non-smokers (Ref.)												
Current smokers	-163.13	80.38	-2.03	0.04	-85.07	38.48	-2.21	0.03	7.79	85.67	0.09	0.9

* Three-category variables were tested by F-test. BMI: body mass index.

## DISCUSSION

This multiple-center retrospective cohort study explored factors associated with GWG stratified by the maternal pregestational weight status (BMI). We found that maternal pregestational BMI and maternal smoking status were significantly associated with higher GWG. Furthermore, our findings supported those of previous studies indicating that maternal smoking during pregnancy is associated with lower birthweight of infants^7-9^.

Our results suggest that most participants followed the GWG guidelines of the Japanese Ministry of Health, Labor and Welfare^15^. However, compared with data from other studies^19^, our GWG data may be lower for each pregestational weight status. Pregestational BMI was significantly associated with lower GWG in normal-weight and overweight categories. Particularly in the overweight category, it was relatively large (b= -0.9). In addition, pregestational BMI showed a trend to be negatively associated with GWG in the underweight category, although this did not reach statistical significance. These findings suggest that pregnant women who smoke need adequate control of their GWG based on pregestational weight status according to the official GWG guidelines^15^.

Maternal smoking status was significantly associated with higher GWG, although maternal smoking during pregnancy was associated with lower birthweight. In contrast, maternal GWG was positively associated with birthweight. These results suggest that among smoking mothers, the effect of maternal smoking during pregnancy on the infant birthweight was greater than the effect of GWG, although GWG was higher in smoking mothers than in non-smoking mothers. It is possible that a proportion of pregnant women quit smoking during pregnancy because information on smoking was obtained during early pregnancy. Smoking cessation has been associated with weight gain^20^. Moreover, maternal smoking during pregnancy may alter metabolic processes, including appetite. In this regard, smoking may be a predisposing factor for abdominal obesity, glucose intolerance, and insulin resistance^21^.

Our previous results suggested that maternal smoking during pregnancy was significantly associated with childhood obesity^22-24^. This association was observed in the second quartile of birthweight^25^. Therefore, the GWG of smoking mothers may compensate for the negative effects of maternal smoking during pregnancy on birthweight. Further studies are warranted to clarify the mechanisms underlying the association between maternal smoking during pregnancy and GWG and the effects of this association on fetal and childhood growth. Moreover, as a matter of course, it is essential to prevent maternal smoking during pregnancy and relapse smoking after delivery. For achievement of these issues, digital evidence-based interventions might be effective, especially during the COVID-19 pandemic^26^.

### Strengths and limitations

The study has several strengths. First, this study was conducted at two community hospitals and one university hospital. Although there were differences in the study periods, the heterogeneity of participants could be a major benefit in this study setting. Moreover, the validity of GWG was relatively high because this study was based on clinical data. Our results highlight the populations who are at high risk of inadequate GWG and may contribute to facilitating interventions for the control of maternal weight during pregnancy. In Japan, the underweight status among young women is a major public health concern. For example, a national health and nutrition survey conducted by the Ministry of Health, Labor and Welfare of Japan indicated that the percentage of underweight women is increasing (13.1% in 1980 to 21.7% in 2017 for people aged 20–29 years; and 7.9% in 1980 to 13.4% in 2017 for people aged 30–39 years)^27^. We believe that our results may help improve the nutrition status among such women.

This study has also some limitations. First, because the study was conducted in a rural Japanese area, we cannot generalize the results, even though this was a multicenter study. Furthermore, owing to limited resources, the study periods for each hospital varied. However, because the primary aim of the study was to identify factors associated with GWG, we reduced the priority of generalization to relatively low. In this regard, our results demonstrate that maternal smoking during pregnancy is negatively associated with birthweight, which is congruent with the results of previous studies^7-9^. Finally, because our study was hospital-based, it was difficult to obtain information on passive smoking, nutrition and physical activity. Further studies are needed to gather relevant lifestyle information to establish statistically appropriate models. In addition, it is important for pregnant women, their partners and close relatives to ensure receiving education about the health risks of active and passive smoking and how these could have an adverse effect to their fetus and infants^28^.

## CONCLUSIONS

This multiple hospital-based retrospective cohort study demonstrates that maternal pregestational BMI and maternal smoking status were significantly associated with higher GWG, and that maternal smoking during pregnancy was associated with lower birthweight of infants. Further studies are warranted to clarify the mechanisms underlying the association between GWG and birthweight, particularly in smoking mothers. In addition, our results may contribute to improving the nutrition status among young women.

## Data Availability

The data supporting this research are available from the authors on reasonable request.
